# Specific serum microRNA profile in the molecular diagnosis of Hirschsprung's disease

**DOI:** 10.1111/jcmm.12348

**Published:** 2014-06-28

**Authors:** Weibing Tang, Hongxing Li, Junwei Tang, Wei Wu, Jingjing Qin, Hao Lei, Peng Cai, Weiwei Huo, Bo Li, Virender Rehan, Xiaoqun Xu, Qiming Geng, Hongwei Zhang, Yankai Xia

**Affiliations:** a State Key Laboratory of Reproductive Medicine, Institute of Toxicology, School of Public Health, Nanjing Medical UniversityNanjing, China; b Department of Pediatric Surgery, Nanjing Children's Hospital Affiliated Nanjing Medical UniversityNanjing, China; c Key Laboratory of Modern Toxicology, Nanjing Medical University, Ministry of EducationNanjing, China; d Department of Pediatric Surgery, Xuzhou Children's Hospital Affiliated Xuzhou Medical UniversityXuzhou, China; e Harbor UCLA Medical Center, David Geffen School of Medicine at UCLATorrance, CA, USA

**Keywords:** HSCR, serum miRNA, diagnosis, signature

## Abstract

Hirschsprung's disease (HSCR), a congenital gastrointestinal disorder, is one of the most common causes of neonatal bowel obstruction. Without an early screening and diagnosis, some patients develop serious complications, such as toxic megacolon or acute enterocolitis. We sought to identify specific serum microRNAs (miRNAs) that can serve as novel early, non-invasive screening signature and then to test their specificity and sensitivity in diagnosing Hirschsprung's disease. We obtained serum samples from 95 HSCR cases and 104 matched controls. An initial screening of miRNA expression was performed through TaqMan Low Density Array. The candidate miRNAs were validated by individual reverse transcription quantitative real-time PCR arranged in the training and a two-stage validation set. Additional double-blind testing was performed in 23 patients with clinically suspected HSCR to evaluate the diagnostic value and accuracy of the serum miRNA profile in predicting HSCR. Following a multi-stage evaluation approach, five miRNAs were significantly increased in HSCR cases compared with controls. The areas under the receiver operating characteristic (ROC) curve of this five-serum miRNA signature were 0.895, 0.893 and 0.925 in training set and two validation sets, respectively. The accuracy rate of the five-miRNA profile as HSCR signature was 82.6%, which, in the double-blind testing set, was markedly higher than that of contrast enema (70%), the most commonly used test performed to diagnose HSCR. Our results indicate that a five-serum miRNA signature may be linked to HSCR, representing a potential, novel, non-invasive diagnostic approach for early screening of HSCR.

## Introduction

Hirschsprung's disease (HSCR), caused by the failure of the enteric neural crest cells (ENCCs) to migrate normally, is one of the most common congenital digestive diseases [1]. The incidence of this disease is about 1:5000 in live births, with males being four times more likely to be affected than females [2]. Most affected individuals have ‘short-segment’ disease where aganglionosis is restricted to the rectosigmoid region of the colon, and ‘long-segment’ disease where aganglionosis extends proximal to the sigmoid colon (confined within the large bowel). A minority of the cases are represented by very short-segment Hirschsprung and total colonic aganglionosis [3,4]. Eighty to ninety per cent of the children are diagnosed in the neonatal period, mostly based on three tests: contrast enema (CE), in which the critical feature to suspect HSCR is the presence of a transitional zone; anorectal manometry (ARM) that assesses the rectoanal inhibition reflex; and rectal suction biopsy (RSB), which shows aganglionosis. Full-thickness rectal biopsy was the original gold standard for the diagnosis of HSCR [5]. While the current study indicates that the diagnosis of HSCR is not always easy to establish, each of these tests has both advantages and disadvantages in availability, technical difficulty, radiation exposure and invasiveness [6,7]. Currently, the only treatment is primarily surgical removal of the aganglionic bowel. Nevertheless, the delayed diagnosis of HSCR increases the risk of complications such as the toxic megacolon or acute enterocolitis, leading to poor long-term outcome [8]. Therefore, more effective and accurate diagnostic approach, especially by using non-invasive diagnostic methods, is needed in early screening and diagnosis of HSCR.

MicroRNAs (miRNAs) are small, non-coding RNA molecules of 19–25 nucleotides that function as regulators of gene expression [9]. The expression of miRNAs in serum, plasma, semen and other body fluids, particularly serum miRNAs, is abundant and stable [10]. We believe that serum-based screening for HSCR will be easier, non-invasive and more affordable than the three tests mentioned above. Recent studies by our group and others have demonstrated that miRNAs are closely related to various diseases, including cancers, male infertility, gestational diabetes mellitus, idiopathic nephrotic syndrome, *etc*. [11–15]. The presence of specific serum miRNAs in other diseases raised the hypothesis that similar miRNAs probably exist in serum of patients with Hirschsprung.

In this study, to identify new miRNA signature for HSCR prediction, we first focused on the miRNA expression profiles in the serum samples of HSCR cases through the TaqMan Low Density Array (TLDA) and reverse transcription quantitative real-time PCR (RT-qPCR) assays, which, in addition to offering a specific expression profile, also provide useful information about the probable molecular pathogenesis of HSCR.

## Materials and methods

### Study design, patients and control volunteers

**Fig. 1:**
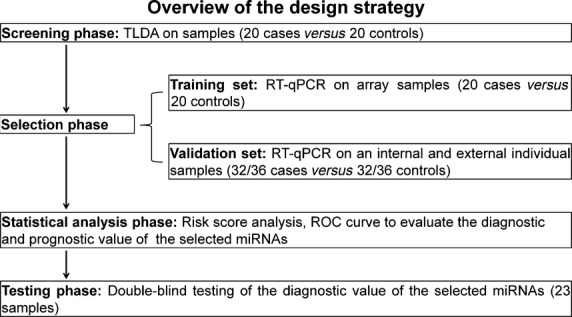
Overview of the design strategy. A multi-stage, case–control study was designed to identify a serum miRNA profile as a signature for HSCR. A screening stage and two validation sets were performed to estimate the expression level of serum miRNAs, risk score analysis, ROC curves, and additional double-blind testing performed to evaluate the diagnostic capability of the candidate miRNAs.

The study enrolled 95 patients with a pathological diagnosis of HSCR and 104 matched controls who were admitted to the department of pediatric surgery, Nanjing Children's Hospital (NJ) and Xuzhou Children's Hospital (XZ), China, between 2012 and 2013. This study was approved by the Institutional Ethics Committee of Nanjing Medical University, and a written informed consent was obtained from the parent of each participant. A multi-stage, case–control study was designed to identify a serum miRNA profile as a signature for HSCR (Fig. 1). In the screening stage, 20 cases and 20 matched control serum samples from NJ were subjected for TLDA to identify the miRNAs that were differentially expressed between HSCR cases and controls. Thereafter, we performed individual RT-qPCR in the training phase to further filter signals of the screened miRNAs. Subsequently, we perfected the number of serum miRNAs included as the HSCR signature by a two-stage validation, including an internal and an external validation. For internal validation, 32 cases and 32 controls from NJ were tested, whereas the external validation phase used serum samples from an additional 36 cases and 36 controls from XZ. We also analysed another seven HSCR cases and 16 controls from NJ in a blinded fashion (the investigators performing the molecular analysis on the blood samples were blinded to the patients' clinical diagnosis) to validate the diagnostic capability of the candidate miRNAs. The protocols, including the diagnosis procedure and serum collection methods, were identical in two hospitals participating in this study.

HSCR diagnosis was confirmed by pathological analysis after surgery. The short-segment and long-segment disease classification was based on operative findings. The matched controls, admitted to hospital, because of abnormal physical examination, history of trauma, suspected neonatal haemangiomatosis and so on, were proven to be without HSCR or other congenital malformations. Another 23 serum samples (7 HSCR cases and 16 controls) were collected following the same approach as outlined before for the double-blind test except that all the patients had the symptom of abdominal distension and clinically suspected HSCR. Moreover, all the patients for the double-blind test had CEs, and the controls were ruled out from having HSCR. Clinical and demographic characteristics of the cases and controls are summarized in Table 1.

**Table 1:** Demographic and clinical features of study subjects

Variable	Control (*n* = 104)	HSCR (*n* = 95)	*P*
Age (months, mean, SE)	3.37 (0.23)	3.70 (0.22)	0.30*
Sex (%)			
Male	80 (76.90)	76 (80.00)	0.60†
Female	24 (23.10)	19 (20.00)	
Classification (%)			
Short-segment		46 (48.00)	
Long-segment		49 (52.00)	

* Student's *t*-test.

† Two-sided chi-squared test.

### RNA isolation and RT-qPCR assay

Venous blood samples from each fasting participant were collected from both cases and controls prior to any therapeutic procedures in a serum separator tube; the gut tissue specimens were also collected, and were stored at −80°C until analysis. Total RNA, containing miRNA, was extracted from blood and tissue specimens by using the TRIzol reagent (Life Technologies, Carlsbad, CA, USA), according to the manufacturer's protocol. The RNA was quantified by using a NanoDrop 2000 Spectrophotometer (Thermo Scientific, Wilmington, DE, USA) and immediately stored at −80°C until analysis.

MiRNA-specific TaqMan MicroRNA Assay (Life Technologies Inc.) was used for the serum miRNAs; the probe information is shown in Table S1. One microgram of the total RNA was reverse-transcribed with the TaqMan MicroRNA Reverse Transcription Kit (Life Technologies Inc.). RT-PCR was performed with the ABI 7900 HT Real-Time PCR System (Life Technologies Inc.). The reactions were initiated in a 384-well optical plate at 95°C for 5 min, followed by 40 cycles of 95°C for 15 sec. and 60°C for 1 min. Expression levels of miRNAs were normalized to miR16 as internal control in concordance with other publications [16]. The data obtained were calculated by the ΔCq method as described previously [17]. The relative expression corresponded to the 2^ΔCq^ value.

### TaqMan low-density array

In the screening stage, TLDA Chips (Life Technologies Inc.) was used to screen differentially expressed miRNAs from the two pooled samples. Megaplex RT reactions and pre-amplification reactions were run according to the manufacture's protocol, in which 75-μl 0.16 * TE was added to PreAmp product, and 9-μl diluted PreAmp product was used to run the RT-PCR reactions by dispensing 100 μl of the PCR reaction mix into each port of the TaqMan MicroRNA Array. The default PCR procedure was used, and the analysis was performed by using RQ manager software (Life Technologies Inc.).

### Statistical analysis

The miRNA data were expressed as the median (interquartile interval), and other variables were expressed as the mean (SD). Chi-squared tests and the Student's *t*-test were used to evaluate statistical differences in demographic and clinical characteristics. The non-parametric Mann–Whitney *U*-test was used to compare differences in serum miRNA expression between HSCR cases and controls. Risk score analysis was performed to investigate the effectiveness of the five-serum miRNA signature for HSCR predicting as described previously [18]. More details about statistical analysis are in supplementary material.

## Results

### Volunteer characteristics

A total of 199 samples were included in this study, among which 95 cases were proven by pathology and classified into 46 short-segment and 49 long-segment HSCR patients. As shown in Table 1, there was no significant difference in the distribution of age and sex between HSCR cases and matched controls. The gender rate (male/female) of HSCR cases and controls was 76/19 and 80/24, respectively.

### Screening and selection phase

To gain an expression profile of serum miRNAs that is specific for HSCR, the TLDA was used to identify the differentially expressed miRNAs in 20 HSCR cases and 20 controls in the initial screening phase. As shown in Table S2, among 667 miRNAs analysed, eight up-regulated miRNAs (miR-133a, miR-451, miR-218-1, miR-628-5p, miR-92a, miR-25, miR-193b and miR-483-5p) were identified, which satisfied the threshold, at most 30 of CT value and at least on average 64-fold change by TLDA, in both two pools, for further individual validation.

We further examined the eight candidate miRNAs by RT-qPCR in a training sample set (the same sample set used in TLDA). As the difference in miR-193b, miR-451 and miR-628-5p expression level between HSCR cases and controls did not reach statistical significance (data not shown) in this assay, these were excluded from further analysis. The remaining five miRNAs (miR-133a, miR-218-1, miR-92a, miR-25, miR-483-5p) remained significantly altered in HSCR samples (see Fig. S1A–E).

**Fig. 2:**
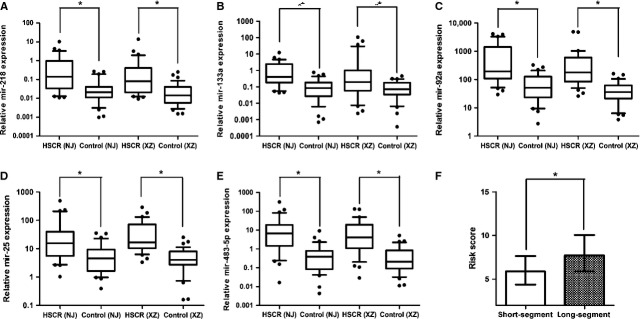
The aberrantly expressed five miRNAs between the HSCR cases and controls in the two validation sets. And, risk score values in short-segment and long-segment HSCR patients. Expression level of the five candidate miRNAs in the serum of HSCR cases and controls (NJ 32 cases *versus* 32 controls, XZ 36 cases *versus* 36 controls) (**A**–**E**). Risk score values in HSCR patients between short-segment and long-segment (**F**). Data are presented as box plot of the median and range of log-transformed relative expression levels. The top and bottom of the box represent the seventy-fifth and twenty-fifth percentiles. The whiskers indicate the 10th and 90th points (* denote *P* < 0.05).

To validate the accuracy and specificity of these five miRNAs as a HSCR potential signature, we also examined their expression levels through internal and external individual samples (NJ, 32 cases *versus* 32 controls, XZ, 36 cases *versus* 36 controls). As shown in Table 2, the expression of five miRNAs in serum of HSCR cases was all significantly higher than that in controls in the two data sets, which was in agreement with the training set results. As a result of this multi-phase testing and analysis, a profile of five miRNAs was considered to be the potential signature for HSCR. The expression level of five miRNAs between the HSCR cases and controls was shown in Figure 2A–E.

**Table 2:** Serum miRNAs differentially expressed in HSCR cases compared with controls*

	Training set			Validation set					
				
				NJ			XZ		
			
	Control	HSCR	*P*	Control	HSCR	*P*	Control	HSCR	*P*
miR-25	0.19 (0.02–0.78)	5.00 (2.00–15.17)	1.70 × 10^−5^	4.54 (1.63–9.27)	15.49 (5.52–39.59)	2.00 × 10^−4^	3.99 (2.70–7.95)	16.57 (10.39–71.57)	4.73 × 10^−9^
miR-92a	4.79 (2.25–16.01)	65.08 (29.66–107.15)	1.00 × 10^−4^	50.91 (23.23–126.48)	194.86 (107.34–1420.25)	2.79 × 10^−6^	35.73 (21.06–62.36)	177.23 (84.31–595.06)	4.42 × 10^−9^
miR-133a	0.015 (0.01–0.05)	0.13 (0.01–0.60)	3.00 × 10^−2^	0.08 (0.03–0.18)	0.40 (0.18–2.41)	1.36 × 10^−5^	0.07 (0.04–0.18)	0.20 (0.06–1.00)	7.86 × 10^−3^
miR-218-1	0.02 (0.01–0.07)	0.16 (0.03–0.21)	1.10 × 10^−3^	0.02 (0.01–0.04)	0.14 (0.04–0.10)	4.73 × 10^−5^	0.02 (0.01–0.04)	0.81 (0.02–0.45)	5.37 × 10^−6^
miR-483-5p	0.23 (0.12–0.69)	1.75 (0.86–15.44)	3.49 × 10^−5^	0.39 (0.09–0.78)	6.61 (1.45–18.75)	1.75 × 10^−6^	0.21 (0.09–0.84)	4.14 (1.09–19.38)	8.28 × 10^−8^

* Data are expressed as the median (interquartile range).

### Diagnostic value of the considered serum miRNAs by risk score analysis

To assess the diagnostic value of the five-serum miRNA profiling system, we used a risk score formula to calculate the risk score function (RSF) for cases and control samples. First, the risk score of each serum sample in the training set was calculated, as the basis of their risk scores and a set cut-off, serum samples were then divided into a high-risk group, representing the possible HSCR cases, and a low-risk group, representing the predicted controls. At the optimal cut-off value (2.94), with the value of sensitivity + specificity considered to be maximal, the diagnostic sensitivity and specificity of the five-serum miRNA signature for HSCR detection were 80% and 95%, and the positive predictive value and negative predictive values were 94% and 83%, respectively, in the training set. Similarly, when the same cut-off of 2.94 was used to calculate the risk score of samples from NJ and XZ validation sets, the diagnostic sensitivity and specificity were 84% and 84% (NJ), and 86% and 83% (XZ) (Table 3), respectively, which were higher than those for CE (70% and 83%) [19], the most common method for the evaluation of children with suspected HSCR.

**Table 3:** Risk score analysis of HSCR cases and controls

Score	0–2.94	>2.94–13	PPV	NPV
Training set				
Control	19	1	0.94	0.83
HSCR	4	16		
Validation set, NJ (XZ)				
Control	27 (30)	5 (6)	0.84 (0.84)	0.84 (0.86)
HSCR	5 (5)	27 (31)		

PPV: positive predictive value; NPV: negative predictive value.

**Fig. 3:**
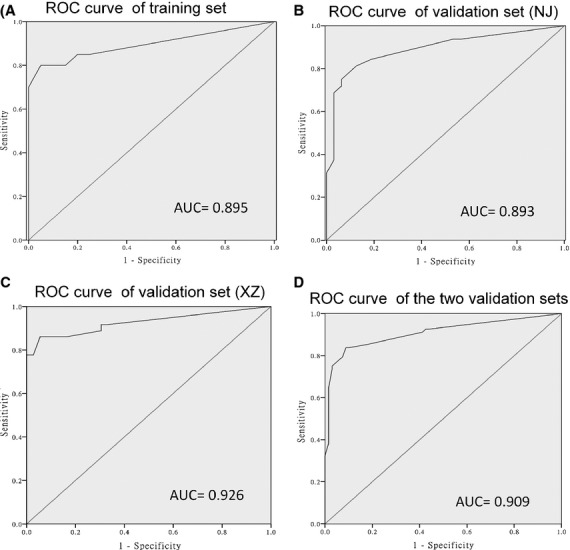
ROC curve analysis for discrimination between HSCR cases and controls by the 5-miRNA signature profile. ROC curve for the 5-miRNA signature to separate 20 HSCR cases from 20 controls in the training set (**A**). ROC curve for the 5-miRNA signature to separate 32 HSCR cases from 32 controls (NJ) and 36 HSCR cases from 36 controls (XZ) in the validation set respectively (**B** and **C**). ROC curve for the 5-miRNA signature to differentiate the HSCR cases and controls in the combined NJ and XZ data set (**D**).

Receiver operating characteristic (ROC) curve analyses were then conducted to assess the diagnostic sensitivity and specificity of the five-serum miRNA signature for HSCR by using RSFs. The areas under the curve (AUC) were 0.895 [95% confidence interval (CI), 0.78–1.00], 0.893 (95% CI, 0.809–0.976), 0.926 (95% CI, 0.859–0.993) and 0.909 (95% CI, 0.856–0.962) for the serum samples in training set, two validation sets (NJ and XZ) and the whole validation set respectively (Fig. 3). These results indicate that the five-serum miRNA signature can serve as a novel non-invasive approach for the early screening of HSCR.

The unsupervised cluster method was also performed to analyse the differential expression of miRNAs between the HSCR cases and controls. The dendrogram generated showed a clear separation of the HSCR cases from the control samples on the basis of the five-serum miRNA signature after the cluster analysis. As shown in Figure S2A, in the training set, only three HSCR case samples and three control samples were misclassified. In the NJ and XZ validation set, only six of 68 HSCR cases and 12 of 68 controls were incorrectly classified (see Fig. S2B).

### Correlation of serum miRNA level with demographic and clinical factors

Furthermore, considering whether the expression of the five-serum miRNA signature was affected by the age, sex and clinical classification, we analysed the correlation of the expression levels of the five miRNAs with these factors by using the non-parametric Mann–Whitney *U*-test. No significant differences in the expression levels of five miRNAs were detected by using age and sex as independent variables; however, the risk score based on the five-serum miRNA signature was progressively higher for long-segment *versus* short-segment HSCR cases (Fig. 2F).

### Validation of the diagnostic capability of the five-serum miRNA signature by a double-blind test

Another 23 serum samples (7 HSCR cases and 16 controls) were tested in a double-blind fashion to validate the predictive ability of the five miRNA–based signature for HSCR diagnosis. We used the same risk score formula to analyse the expression of the five miRNAs in those serum samples and classify them into a high-risk group and a low-risk group. On the basis of the pathological diagnosis, the accuracy rate of the five-miRNA profile as HSCR signature was 82.6%, which is higher than that of CE (70%), however, lower than of RSB, for the same sample set (see Table S3).

## Discussion

HSCR is a common malformation of the digestive tract of the newborn. Diagnosis of HSCR relies mainly on clinical manifestations such as intestinal obstruction, failing to pass meconium, abdominal distension in the neonatal period, and subsequent diagnostic tests by using various tests, including CE, ARM and RSB. Some diagnoses of HSCR are missed because of atypical clinical signs in the neonatal period. Commonly, CE radiographs of the colon are normal for the first few months of life and, in fact, indefinitely in patients with total colonic disease [20]. A ‘transition zone’ may be visible on a CE radiograph; however, transition zone does not reliably delineate aganglionic bowel [21]. Furthermore, the sensitivity (70%) and specificity (50–80%) of diagnosing HSCR by using CE are considerably lower than other methods [6,19]. The ARM demonstrates the absence of internal anal sphincter relaxation upon rectal distention; however, in infants less than 14 weeks of age, the false positivity is too high as the internal sphincter reflex is rudimentary because of its immature nerve supply [22]. Moreover, the ARM requires expensive equipment, patient's cooperation and significant experience of technical expertise. Another frequently used diagnostic test, RSB, which is the most accurate method, involves taking small pieces of the rectal mucosa, with a sensitivity and specificity of 93% and 98% respectively [19]. Nevertheless, from a practical standpoint, the harvested specimens must be taken 1, 3 and 5 cm above the pectinate line and should contain enough submucosa to properly assess the ENS, which increase the probability of serious bleeding and bowel perforation [23,24]. At present, the most reasonable and early screening strategies of HSCR are still unclear, leading to different approaches in different hospitals, with frequent poor functional outcome. Therefore, better, safer and more effective, especially, non-invasive diagnostic strategies are needed for early HSCR diagnosis.

Several previous studies have reported that the unique serum miRNA expression profiles for various diseases may serve as fingerprints for disease detection [25]. A serum miRNA–based signature would make it possible to comprehensively and non-invasively diagnose HSCR, either without or with decreased use of RSB and other invasive procedures. Supporting our approach, specific miRNAs have been found in the central neural system during embryo development [26,27]. To our knowledge, this is the first report that demonstrates the use of serum miRNA profile as a potential biomarker of paediatric HSCR. We identified five HSCR-associated miRNAs, including miR-25, miR-92a, miR-133a, miR-218-1 and miR-483-5p. Our results indicate that HSCR is associated with a combination of multiple serum miRNAs, providing a comprehensive indicator for its detection. Logistic regression analyses and ROC curves revealed a strong relationship between these miRNAs and HSCR, with an accuracy rate of the five-serum miRNA profile being 82.6%, which was higher than that of CE (70%) in the double-blind test. Our results show that the expression profile of the five identified serum miRNAs can serve as a novel non-invasive approach for early screening of HSCR.

Not only will such information provide a serum miRNA profile for molecular diagnosis and assessment in HSCR but will also provide novel mechanistic insight into the pathogenesis and progression of this disease. Our study, by implicating miRNAs involved in cell differentiation, proliferation, migration and apoptosis of the ENCCs, suggests a potential novel pathogenetic mechanism for HSCR. To date, more than 10 genes have been identified to be associated in the pathogenesis of HSCR. *RET* proto-oncogene, known as tyrosine kinase receptor, is widely expressed in neural crest cell, which is crucial for the development of enteric neurons and plays an important role in the pathogenesis of HSCR [28]. Among the five serum miRNAs identified in the HSCR patients by us, miR-218-1 has been shown to be the target miRNA of RET [29], involved in cell migration and proliferation [30]. However, the precise mechanisms by which the five miRNAs, especially the miR-218-1, and their target genes regulate HSCR progression remain unclear. Further studies are required to identify the function and target genes of the five serum HSCR–related miRNAs and the mechanism that regulates the biogenesis of these miRNAs.

In the present study, we identified a clear correlation between serum miRNA expression and the clinical classification of HSCR. Given that the clinical classification is important tool used by paediatricians to predict prognosis and determine treatment, five-serum miRNA profile can be immensely useful in clinical phenotyping and in deciding optimal therapeutic strategies for HSCR patients.

Previous studies have described that miRNAs are not derived only from circulating blood cells but also from tissues affected by the disease [15,31]. A comparison of the miRNA expression patterns in serum and tissue may provide additional evidence supporting the use of serum miRNAs as reliable diagnostic biomarkers. We identified significantly increased levels of miR-218-1 and miR-483-5p in both serum and tissue samples from HSCR patients (see Fig. S3), which indirectly imply this conclusion that these serum miRNAs could be derived from HSCR tissues.

When considered as a single diagnostic method, the five-serum miRNA signature indicated a high sensitivity and specificity, significantly higher than that of CE. However, the expression level of miRNAs in the validation set (both the NJ group and the XZ group) shares the overlapping values with training set. We re-investigated the expression level of the miRNAs in the training set; the results indicated no statistical difference with the former data as we presented in Table 2. Because of a small sample size in the training set, we thought it is possible to obtain overlapping values between the training set and the validation set. In addition, the RNA samples in training set used for RT-PCR assay were frozen and thawed for the second time after the microarray detection; thus, it might caused degradation of the RNA. Therefore, a larger sample size is needed to validate the diagnostic capability of the five-serum miRNA signature. Moreover, given the expression fluctuations of individual serum miRNAs, analysis with a panel of multiple diagnostic methods might be a more useful approach. For example, the combination of CE and five-serum miRNA signature may further improve the diagnostic accuracy for HSCR. To test this, more cases of the HSCR, tested by both the five-serum miRNA signature and CE, are required.

In conclusion, we have identified a unique five-serum miRNA signature for early screening of HSCR, which may serve as a novel non-invasive approach for diagnosis of HSCR.

## Supporting information

**Figure S1** The expression level of the five-serum miRNA signature in HSCR cases and controls.

**Figure S2** Cluster analysis of the miRNA differentially expressed between HSCR and control serum samples.

**Figure S3** Comparison of the expression levels of 5 miRNAs in HSCR and control gut samples.

**Table S1** AB assay ID of the miRNAs used in the research. **Table S2** Up-regulated miRNAs in serum samples from HSCR compared to controls determined by TLDA. **Table S3** The result of the double-blind test.
