# Supplementary figures and images for: Specific serum microRNA profile in the molecular diagnosis of Hirschsprung's disease
Source: J Cell Mol Med. 2014 Jun 28;18(8):1580–7. doi: 10.1111/jcmm.12348 (PMC4190904; doi:10.1111/jcmm.12348)

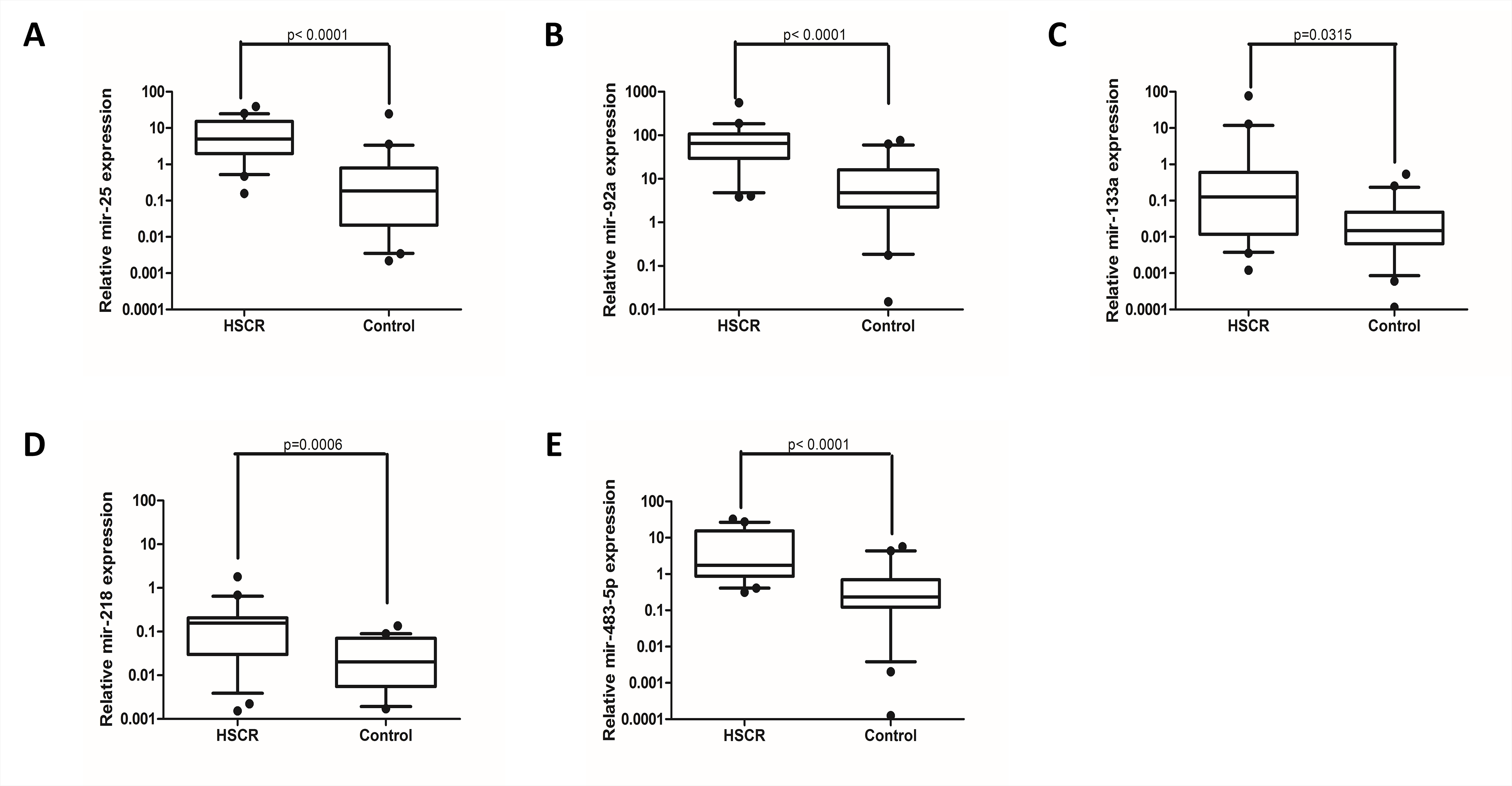

Supplement: Supplementary file 1 — Figure S1 The expression level of the five-serum miRNA signature in HSCR cases and controls. [file jcmm0018-1580-SD1.tif]

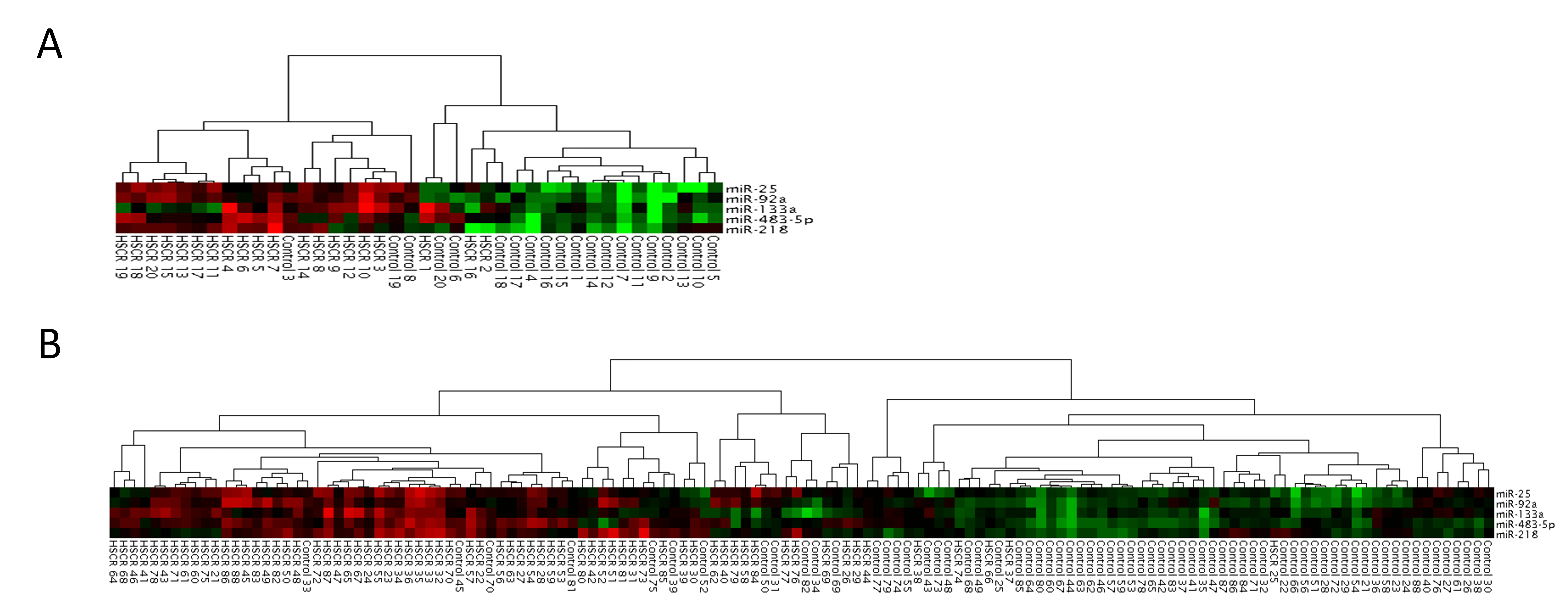

Supplement: Supplementary file 2 — Figure S2 Cluster analysis of the miRNA differentially expressed between HSCR and control serum samples. [file jcmm0018-1580-SD2.tif]

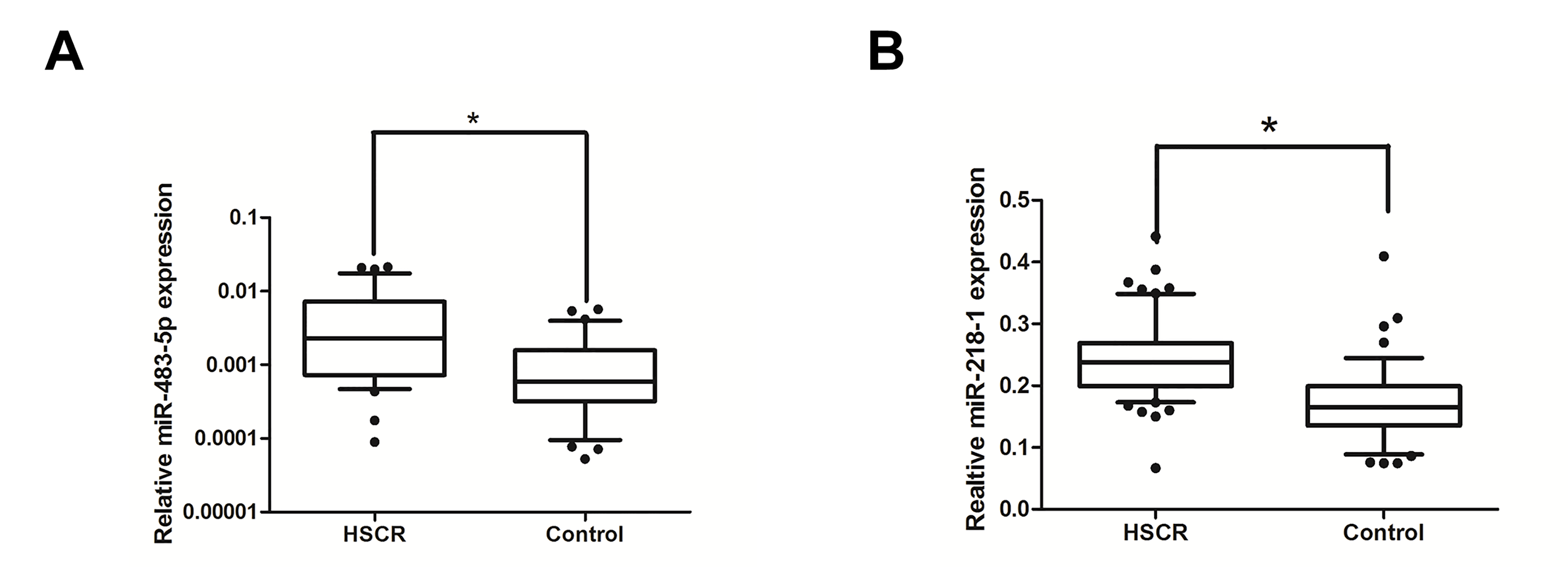

Supplement: Supplementary file 3 — Figure S3 Comparison of the expression levels of 5 miRNAs in HSCR and control gut samples. [file jcmm0018-1580-SD3.tif]
