# Supplementary material for: Specific serum microRNA profile in the molecular diagnosis of Hirschsprung's disease
Source: J Cell Mol Med. 2014 Jun 28;18(8):1580–7. doi: 10.1111/jcmm.12348 (PMC4190904; doi:10.1111/jcmm.12348)
Supplement: Supplementary file 4 [file jcmm0018-1580-SD4.doc]

**Supplementary Online Content**

**Specific Serum MicroRNA Profile in the Molecular Diagnosis of** **Hirschsprung’s Disease**

*Statistical analysis*

The serum miRNA expression are presented as box plot of the median and range of log-transformed relative expression level between HSCR cases and controls. Risk score analysis was performed to evaluate the associations between the concentrations of the serum miRNAs expression levels and HSCR. The upper 95% reference interval of each miRNA value in controls was set as the threshold to code the expression level of the corresponding miRNA for each sample as 0 and 1 in the training set. A risk score function (RSF) to predict HSCR was defined according to a linear combination of the expression level for each miRNA. For example, the RSF for sample i using information from five miRNAs was: rsf_i_=∑^5^_j-1_W_j_.s_ij_. In the above equation, s_ij_ is the risk score for miRNA j on sample i, and W_j_ is the weight of the risk score of miRNA j. The risk score of five miRNAs was calculated using the weight by the regression coefficient that was derived from the univariate logistic regression analysis of each miRNAs. Samples were ranked according to their RSF and then divided into a high-risk group, representing the predicted HSCR cases, and a low-risk group, representing the predicted control individuals. Frequency tables and ROC curves were then used to evaluate the diagnostic effects of the profiling and to find the appropriate cutoff point. Frequency tables and ROC curves were then used to evaluate the diagnostic effect of the profiling, to find the appropriate cutoff point, and to validate the procedure and cutoffs in the next validation sample set. Statistical analysis was performed using STATA 9.2, and presented with GraphPad Prism 5.0 software. Results were considered statistically significant at *p* < 0.05.

**Supplementary Tables**

**Supplementary Table 1:** AB assay ID of the miRNAs used in the research.

**Supplementary Table 2:** Up-regulated miRNAs in serum samples from HSCR compared to controls determined by TLDA.

**Supplementary Table 3:** The result of the double-blind test.

**Supplementary Table 1**

AB assay ID of the miRNAs used in the research

| MiRNA | Assay type | AB assay ID |
| --- | --- | --- |
| hsa-miR-133a | Mature miRNA | 002246 |
| hsa-miR-451 | Mature miRNA | 001141 |
| hsa-miR-218 | Mature miRNA | 000521 |
| hsa-miR-92a | Mature miRNA | 000431 |
| hsa-miR-193b | Mature miRNA | 002367 |
| hsa-miR-628-5p | Mature miRNA | 002433 |
| hsa-miR-483-5p | Mature miRNA | 002338 |
| hsa-miR-25 | Mature miRNA | 000403 |
| Has-miR-16 | Mature miRNA | [000391](https://products.appliedbiosystems.com:443/ab/en/US/adirect/ab?cmd=ABAssayDetailDisplay&assayID=000391&Fs=y&SearchRequest.Common.PageNumber=1&assayType=taqman&chkBatchQueryText=false&srchType=keyword&searchValue=miR-16&searchBy=all&msgType=ABmiRNAKeywordResults) |

**Supplemental Table 2**

**Up-regulated miRNAs in serum samples from HSCR compared to controls determined by TaqMan Low Density Assay.**

| MiRNA | HSCR  (Cq value) | Controls  (Cq value) | Fold change (HSCR/Controls) |
| --- | --- | --- | --- |
| hsa-miR-133a | 24.602 | 29.311 | 813.50 |
| hsa-miR-451 | 24.858 | 27.631 | 212.60 |
| hsa-miR-218 | 25.331 | 27.812 | 173.65 |
| hsa-miR-628-5p | 25.975 | 28.079 | 133.71 |
| hsa-miR-92a | 23.719 | 25.736 | 125.89 |
| hsa-miR-25 | 25.509 | 27.288 | 106.74 |
| hsa-miR-193b | 27.061 | 28.724 | 98.50 |
| hsa-miR-483-5p | 25.877 | 27.153 | 75.32 |

**Supplementary Table 3**

**The result of the double-blind test.**

|  |  | HSCR (n = 7) ^a^ | Other diseases (n = 16) ^b^ | Accuracy rate ^c^ |
| --- | --- | --- | --- | --- |
| pathologic diagnosis  rectal suction biopsy(RSB) |  | 7  7 | 16  16 | 100%  100% |
| serum 5-miRNA biomarker  contrast enema(CE) |  | 6  4 | 13  12 | 82.6%  70% |

^a^ HSCR-positive

^b^ HSCR-negative

^c^ Accuracy rate = (HSCR-positive + HSCR-negative) / total

**Supplementary Figures**

**Supplementary Figure 1.** The expression level of the five-serum miRNA signature in HSCR cases and controls. A-G**)** Differential expression of the 5 miRNAs in the serum of HSCR cases (n = 20) compared to that in controls (n = 20). The expression levels of the five serum miRNAs were measured by TaqMan probe-based RT-qPCR. Data were presented as box plot of the median and range of log-transformed relative expression level. The top and bottom of the box represent the seventy-fifth and twenty-fifth percentile. The whiskers indicate the 10th and 90th points. (*, *P <* 0.05)

**Supplementary Figure 2.** Cluster analysis of the miRNA differentially expressed between HSCR and control serum samples. (A) For training set (20 cases and 20 controls), the expression values of serum miRNAs in HSCR cases and controls were normalized, mean-centered, clustered and plotted as a heat map. (B) Cluster analysis of the miRNA differentially expressed between HSCR cases and controls in the validation set (68 HSCR cases and 68 controls).

**Supplementary Figure 3.** Comparison of the expression levels of 5 miRNAs in HSCR and control gut samples. (A and B) The relative expression of miRNAs (HSCR vs. control), normalized to U6, was assessed using the RT-qPCR assay (n = 32, A: miR-218-1, B: miR-483-5p, the others were not shown). Data are presented as box plot of the median and range of log-transformed relative expression levels. The top and bottom of the box represent the seventy-fifth and twenty-fifth percentiles. The whiskers indicate the 10th and 90th points (*, *P <* 0.05).
